# Associations of infectious disease-specific, electronic, and general health literacy among high school students with prevalent health challenges: a cross-sectional study

**DOI:** 10.3389/fpubh.2025.1613375

**Published:** 2025-05-13

**Authors:** Jie Qin, Yan Gong, Ruijuan Hu, Yifang Lou, Xiaoyan Xuan, Peng Wang, Guangming Gong

**Affiliations:** ^1^Department of Neurology, The First Affiliated Hospital of Zhengzhou University, Zhengzhou, China; ^2^Class 1 of Grade 2023, Sino-American Program, International Department, Zhengzhou No. 7 High School, Zhengzhou, China; ^3^Biology Teaching Group, International Department, Zhengzhou No. 7 High School, Zhengzhou, China; ^4^Class of 2027, South Pasadena High School, South Pasadena, CA, United States; ^5^Department of Microbiology and Immunology, College of Basic Medical Sciences, Zhengzhou University, Zhengzhou, China

**Keywords:** adolescent health literacy, health risk behaviors, eHealth literacy, infectious disease-specific health literacy, general health literacy

## Abstract

The interplay of infection-specific (IDSHL), electronic (eHL), and general health literacy (HL) in shaping adolescent health disparities during recurrent infections remains underexplored. This cross-sectional study mainly evaluated the levels and associations of IDSHL, eHL and HL among 10th–11th graders (*n* = 272) using validated instruments (IDSHLS, eHEALS, HLS-SF12) via anonymous questionnaires. Results revealed that 89.7% of participants reported prevalent health concerns, most notably mood (52.2%), sleep (51.8%), vision (47.8%), weight (34.5%) and gastrointestinal (28.3%) issues, with 66.5% engaging in risk behaviors such as physical inactivity (50.4%) and poor dietary habits (40.4%), while electronic new media overwhelmingly dominated health information acquisition. Literacy proficiency varied: 85.29% exhibited high IDSHL (mean ± SD: 23.23 ± 7.69), 51.1% in high eHL (28.22 ± 8.10) and 48.9% in high HL (34.81 ± 10.29). Binary logistic regression identified IDSHL as an independent HL predictor (OR = 10.28, 95% CI 1.79–59.14, *p* < 0.01) and revealed reciprocal eHL-HL associations (eHL → HL: OR = 23.31; HL → eHL: OR = 23.15; both *p* < 0.01). These findings highlight adolescents’ disproportionate health burdens, digital-focused information acquisition, literacy gaps, and preliminary evidence of a reciprocal IDSHL-eHL relationship within health literacy frameworks. The study advocates integrated interventions combining infection-specific education, digital health training, and behavior modification to address adolescent health disparities in prevention-focused digital healthcare systems.

## 1 Introduction

The global health paradigm is undergoing a fundamental transformation from reactive, disease-centric models to predictive and preventive frameworks, driven by unprecedented advancements in digital health technologies, artificial intelligence (AI), and parallel socioeconomic development. In this evolving context, population health literacy (HL)—defined as the integrated capacity to access, evaluate, and apply health information for evidence-based decision-making (1–3)—has emerged as a critical determinant of health outcomes, surpassing traditional factors such as socioeconomic status and genetic predisposition (1, 3, 4). Longitudinal studies link HL-competent behaviors with apparent reductions in all-cause mortality through improved chronic disease management, vaccination adherence, and more effective use of health services (5, 6). Moreover, robust HL is associated with lower risks of emotional disturbances, physical decline, obesity, and both chronic and infectious diseases, ultimately contributing to extended disability-adjusted life expectancy (7, 8).

The global resurgence of respiratory pathogens since 2023—notably influenza A (H1N1), evolving SARS-CoV-2 variants, and *Mycoplasma pneumoniae*—has exposed persistent systemic vulnerabilities in public health infrastructure, reinforcing the imperative for integrated HL frameworks in outbreak preparedness (9). Conventional health education models have proven increasingly inadequate in digitally saturated societies, particularly when addressing the cognitive developmental stages of adolescents and practical implementation constraints. In response, modern HL frameworks have developed two specialized dimensions: e-Health Literacy (eHL) for digital health ecosystem navigation (10–14) and Infectious Disease-Specific Health Literacy (IDSHL) for pathogen-focused risk mitigation (15). Supplementing traditional HL establishing foundational health knowledge, eHL equips individuals with essential digital competencies such as verifying source credibility, critically appraising health information, and efficiently using emerging technologies like AI-driven telemedicine platforms (10–14, 16). Together, HL and eHL form a dual-axis empowerment framework that addresses challenges ranging from health resource disparities, digital divides to information overload (16, 17).

The IDSHL further augments this framework by emphasizing the ability to access, understand, and apply information specific to infectious diseases (15). This specialization is operationalized through recognizing epidemiologic transmission patterns, implementing evidence-based prevention strategies, developing therapeutic intervention literacy, and optimizing adherence to public health guidelines (15). Empirical evidence demonstrates that populations with higher IDSHL scores experience reduced infection rates and improved compliance with non-pharmaceutical interventions during pandemics (15). The integration of HL, eHL, and IDSHL thus establishes a multidimensional framework that synergizes foundational health knowledge, digital adaptability, and pathogen-specific expertise—a critical approach for risk assessment, leveraging technological innovations, and executing precise interventions amid recurrent outbreaks and fragmented health systems (12, 16, 17).

Despite extensive research on HL, the field continues to grapple with a lack of standardized assessment criteria and evaluation tools, leading to persistent methodological heterogeneity (4, 12). To address this shortcoming, our study employs three psychometrically validated instruments: the IDSHL Scale (IDSHLS), culturally validated for Mandarin-speaking populations to assess outbreak-specific knowledge and preventive behavior intentionality (15); the 8-item eHL Scale (eHEALS) (11, 13, 18), a cross-culturally adapted measure for evaluating digital health information appraisal across multinational adolescent cohorts (12, 13, 18); and the 12-item Short-Form Health Literacy Scale (HLS-SF12) (3), a concise general HL measure optimized for adolescent populations with a reduced administration time (3, 13, 18). All instruments are available in Simplified Chinese (SC) and were selected for their high reliability (*α* ≥ 0.85), cross-cultural validity, and practical utility in diverse healthcare settings. The adoption of these specific scales represents a strategic approach to harmonize HL measurement while maintaining operational feasibility for clinical and public health applications (3, 13, 18). This methodological framework not only addresses the current limitations in HL assessment but also provides a standardized approach for comparative studies across different populations and HL domains.

High school students represent a pivotal yet understudied cohort in HL research (4, 18). This group is characterized by (1) health autonomy amid cognitive development, yielding risk appraisal variability; (2) pervasive social media use that enhances eHL while increasing misinformation risk; and (3) educational settings underutilized for IDSHL despite transmission risk (4, 18). The convergence of biological vulnerability, digital immersion, and institutional exposure highlights the urgency of investigating the interplay among HL, eHL, and IDSHL. Such research holds particular urgency for developing school-based interventions capable of dual pandemic mitigation and health equity promotion. Mechanistic understanding of these multidimensional interactions through standardized assessment frameworks is prerequisite for designing context-specific strategies that optimize adolescent health outcomes in high-risk environments (4, 12, 17).

Research on HLs—including general HLS, eHLS, and IDSHLS aspects—has mainly focused on demographic, socioeconomic, and behavioral determinants or on the isolated impacts of individual domains (e.g., eHLS) on HLS and health outcomes (14, 17). However, integrated analyses of these interrelationships remain scarce, particularly among adolescents during active health crises (4, 12, 16, 17). Although standardized tools evaluate each literacy separately, many studies overlook the complex integration required to address real-world socioeconomic, behavioral, technological, and health challenges (18). In practice, effective health decision-making synthesizes general knowledge, digital skills, and pathogen-specific competencies—a dynamic interplay especially critical during outbreaks. This gap constrains our understanding of how these literacies collectively shape adolescent health behaviors and outcomes, a group disproportionately exposed to digital health resources and emerging pathogens (4).

To address these challenges, we conducted a real-time field study among high school students during active outbreaks, using anonymized multidimensional questionnaires to assess prevalent health concerns, demographic–behavioral profiles, primary information channels, and current competencies in IDSHL, eHL, and HL, along with their interrelationships. This approach preliminarily identifies modifiable mediators of literacy integration during cyclical epidemics, thereby establishing an empirical foundation for targeted interventions that enhance adolescent well-being and institutional resilience.

## 2 Methods

Ethical approval for this investigation was obtained from Zhengzhou University Ethics Committee (ZZUIRB-2022-40). During infectious disease resurgence of COVID-19, mycoplasma, and influenza in 2024 (January–November), we administered anonymous paper questionnaires to 300 grades 10–11 students at an international department public high school in Zhengzhou, central China. School and student selection relied on administrative feasibility, with representative adolescent populations underexplored. Exclusion criteria comprised absence during data collection and incomplete survey responses. Grade 12 students were excluded due to university application preparation constraints affecting survey participation rates. The questionnaire’s first page contained an informed consent form detailing study objectives and procedures. Participation was voluntary and anonymous, with completion of the questionnaire serving as implied consent. This approach ensured ethical compliance and facilitated the collection of reliable HL-related data among the students.

### 2.1 Questionnaire design

#### 2.1.1 Sociodemographic information

The questionnaire’s sociodemographic section had variables like sex, ethnicity, residential location, academic year, parental education level and annual household disposable income. They were chosen to capture factors influencing high school students’ HL levels.

#### 2.1.2 Physical condition

Physical condition covers questions on past year’s infectious diseases, recent health events, self-assessment of past year’s health and types of health disturbances in the last year.

#### 2.1.3 Health behavior

Health behavior involves identifying health-risk behaviors, choosing BMI category, self-assessing physical activity level, selecting exercise frequency, and picking daily eating habit option.

#### 2.1.4 Health information

It includes areas of frequent concern (like diet, exercise, etc.), health information channels, types of health education articles often read, and satisfaction level with health information.

#### 2.1.5 Self-evaluation

It involves rating satisfaction with academic performance, interpersonal relationships, and assessing self-efficacy level for achieving goals and overcoming challenges.

#### 2.1.6 HL scales

##### 2.1.6.1 IDSHLS

The IDSHLS, adapted from the Chinese Resident Infectious Disease Health Literacy Scale, was used to measure infectious disease-specific HL (15). It has 22 items and totals 38.62 points. A score of 16.74 or more indicates high HL (15). Its four dimensions are: (1) basic knowledge and concepts of infectious diseases (max 13.17), (2) prevention (max 9.96), (3) management and treatment (max 7.81), (4) pathogen and infection source identification (max 7.68). The overall Cronbach’s *α* is 0.832, with dimension-specific values of 0.652, 0.672, 0.599, 0.632. Each item’s content validity index is ≥ 0.8, ensuring reliability and validity. Permission for using this and related scales was got from the original authors.

##### 2.1.6.2 SC-eHEALS

The eHEALS was initially developed by Norman and Skinner (11) and Barbati et al. (12). Guo et al. (18) adapted the scale into SC and conducted exploratory research among high school students in Henan and other regions of China. Extensive subsequent studies have confirmed the scale’s strong reliability and validity, establishing it as one of the most widely utilized tools for assessing eHL (13). The eHEALS comprises eight items, categorized into three dimensions: the ability to apply online health information and services (items 1–5), the ability to evaluate such information (items 6–7), and the ability to make informed decisions (item 8). Each item is scored on a five-point Likert scale, ranging from “strongly disagree” (1) to “strongly agree” (5). The total score is derived by summing the scores of all items. In this study, scores greater than or equal to the mean are classified as “high level,” while those less than the mean are designated as “low level” (14) (Table 1). The Chinese version of the eHEALS demonstrates excellent internal consistency, with a Cronbach’s *α* coefficient of 0.913, and factor loadings ranging from 0.692 to 0.869.

**Table 3:** Descriptive statistics of IDSHLS, eHEALS, and HLS.

	*N*	Minimum value	Maximum value	Mean value	Standard deviation
IDSHL	272	0.00	38.62	23.23	7.69
eHEALS	272	0.00	40.00	28.22	8.10
HLS	272	0.00	50.00	34.81	10.29
Number of valid cases (column)	272				

##### 2.1.6.3 SC-HLS-SF12

The HLS-SF12, developed by Duong et al. and adapted into SC by Sun et al. (3), is a tool for assessing public HL, applicable to diverse populations including those in Asian regions. It measures three dimensions: HC-HL, DP-HL, and HP-HL. It includes 12 items rated on a 4-point Likert scale (1 = “very difficult” to 4 = “very easy”). The health literacy index is calculated as (mean − 1) * (50/3), with scores ranging from 0 to 50; higher scores indicate greater health literacy. The Chinese version, adapted for cultural and linguistic relevance, shows strong reliability and validity, with a Cronbach’s Alpha of 0.94 for the total scale and 0.86–0.87 for subscales. Test–retest reliability is 0.89. In this study, scores ≥ the mean are classified as “High level,” while scores < the mean are “Low level” (Table 1).

### 2.2 Quality control

The study implemented rigorous data collection protocols with informed consent and purpose clearly outlined in questionnaires. Standardized instructions emphasized response authenticity and data anonymity during small-group sessions (≤30 students) with ≥30-min completion windows. Culturally adapted items ensured comprehension parity, requiring mandatory completion under researcher supervision. Real-time quality control identified and excluded inconsistent/incomplete responses. Dual-researcher electronic data entry and independent statistical analyses ensured reliability.

### 2.3 Statistical analysis

Data analysis was conducted using SPSS software (version 28.0, IBM SPSS Statistics). Count data were expressed as frequencies and percentages (%). The Pearson *χ*^2^ test (chi-square test) was used to compare differences in the distribution of HL categorical variables across demographic and behavioral characteristics, such as sex, grade, urban/rural residence, and physical activity level. Binary logistic regression was employed to assess the influence of these characteristic variables on the binary outcomes of HL levels (high or low). A significance level of 0.05 was set for all statistical tests.

## 3 Results

### 3.1 Demographic and behavioral characteristics

Demographic characteristics of the 272 participants comprised predominantly urban males (55.15, 92.65% urban), with 56.25% in 10th grade. Parental education was predominantly university degrees (78.68%), while household incomes skewed high (67.28%). Behavioral patterns indicated extensive digital engagement (43.38% > 2 h/day) and notable health burdens: 59.19% reported prior-year infections, 24.63% recent illness/injury, and BMI extremes (4.41% obese, 17.28% underweight). Physical activity was suboptimal (45.59% inadequate), coupled with erratic eating patterns (48.53% spontaneous vs. 24.63% balanced). Health information engagement focused on lifestyle topics (31.62%) over disease prevention (8.46%), with 27.21% reporting no health-related reading. Dissatisfaction emerged in health information (12.50%), academics (24.63%), and relationships (18.75%), alongside moderate self-efficacy (44.85%) (Table 1).

### 3.2 Main health issues and health-risk behaviors among high school students

**Figure 1:**
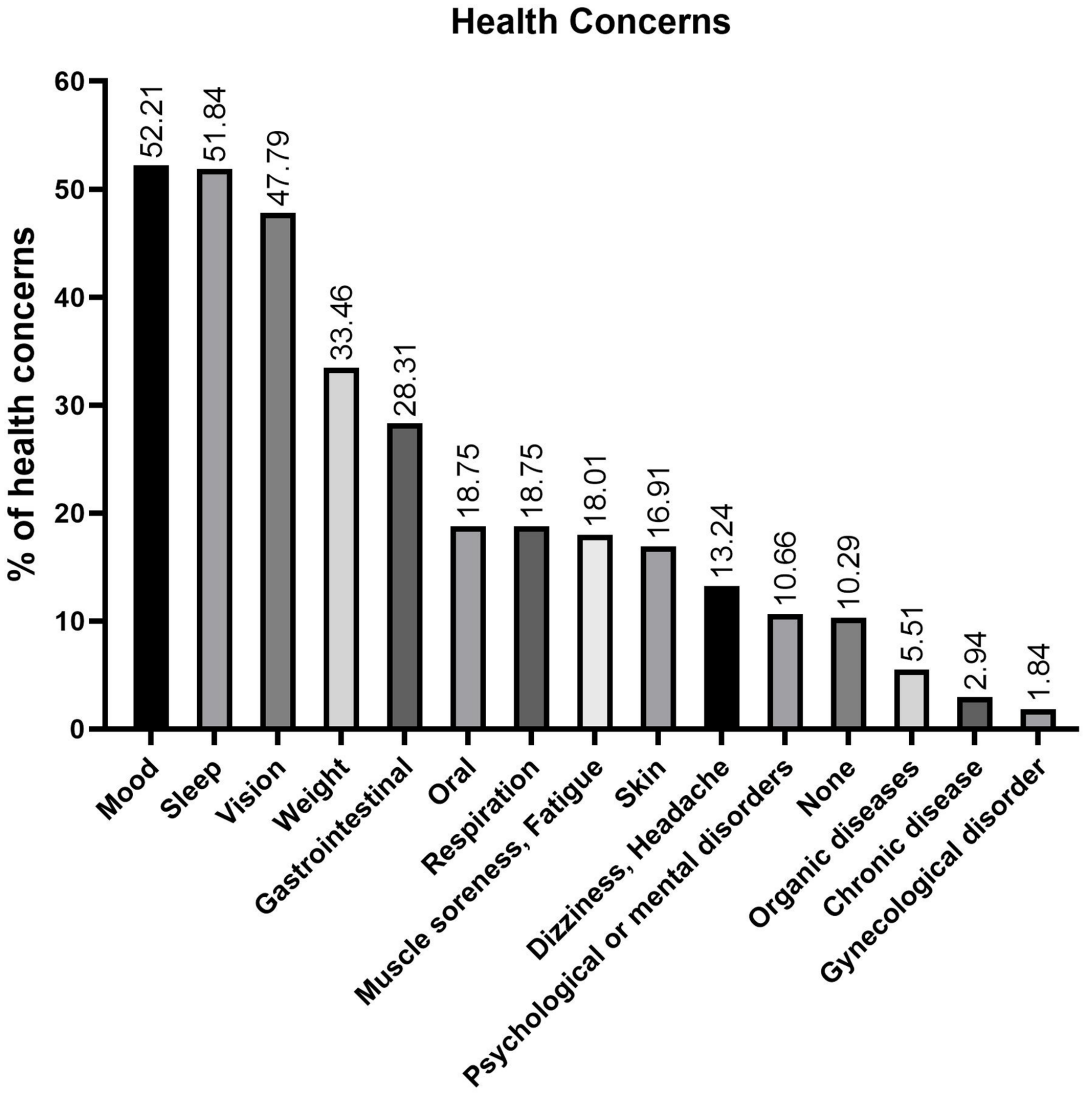
Prevalence of health concerns among senior high school students. The most common issues are mood (52.2%), sleep (51.8%), vision (47.8%), weight (33.5%), and GI (28.3%) problems, with nearly 90% reporting at least one concern, reflecting a 9:1 ratio.

The study revealed near-universal health concerns among high school students, with 89.71% reporting ≥1 issue. Prevalent challenges included mood (52.21%), sleep (51.84%), vision (47.79%), weight (33.46%), and gastrointestinal (GI) (28.31%) disturbances. Respiratory issues affected 18.8%, while only 10.29% reported no health problems (Figure 1).

**Figure 2:**
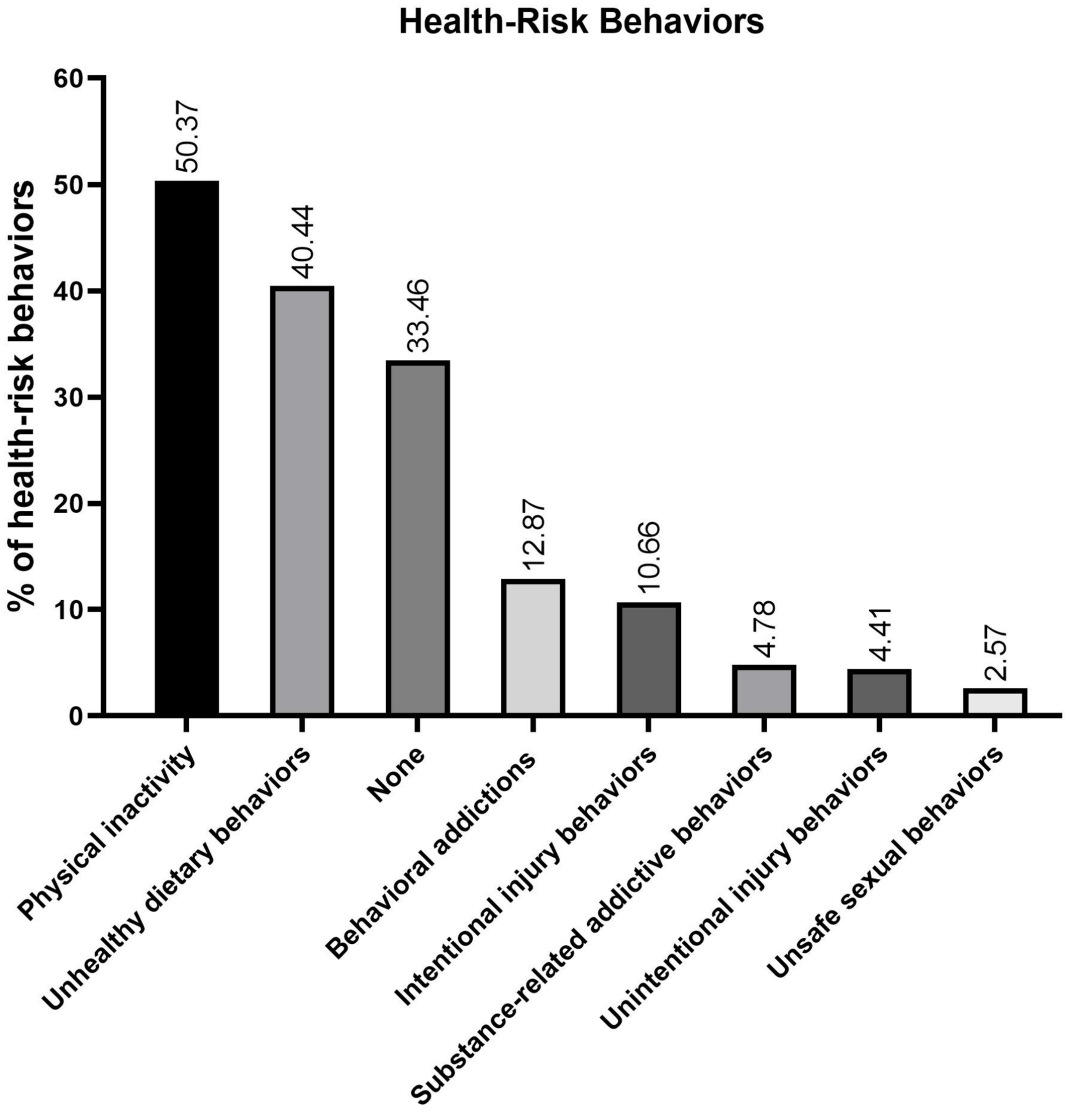
Prevalence hierarchy of health-risk behaviors among senior high school students. Sedentary behavior (50.4%) and suboptimal dietary patterns (40.4%) constitute primary behavioral risks, with 66.5% of participants exhibiting ≥1 risk factor. Non-engagement prevalence reaches 33.5%, establishing a 2:1 risk-to-non-risk ratio.

In terms of health-risk behaviors, physical inactivity (50.37%) and unhealthy eating habits (40.44%) were the most commonly reported. Notably, only 33.46% of students reported engaging in neither behavior, indicating that approximately two-thirds participate in at least one health-risk behavior (Figure 2). These findings underscore the prevalence of common health challenges and risk behaviors among high school students, highlighting the need for targeted interventions to address these issues and promote overall well-being.

### 3.3 Lack of physical activity and unhealthy eating behaviors

The findings revealed that insufficient physical activity is the most prevalent health-risk behavior among senior high school students (Figure 2). Additionally, 45.59% self-identified as generally underactive. In terms of exercise frequency, 15.07% engaged in physical activity less than once weekly, while 48.53% exercised only 1–2 times per week (Table 2). Collectively, these results demonstrate that nearly half of the students fail to meet recommended physical activity levels.

**Table 2:** Responding characteristics of respondents and the association to IDSHL, eHL, HL (*N* = 272).

Characteristics	*N*(%)	IDSHLS			eHEALS			HLS-SF12		
		≥16.74	<16.74	*X* ^2^	High	Low	*X* ^2^	High	Low	*X* ^2^
Daily online time				3.811			1.454			1.693
1. Less than 1 h	71(26.10)	63	8		32	39		35	36	
2. 1 to 2 h	83(30.51)	74	9		45	38		45	38	
3. More than 2 h	118(43.38)	95	23		62	56		53	65	
Any infectious diseases in the past year				0.213			4.172*			3.634
1. Yes	161(59.19)	136	25		74	87		71	90	
2. No	111(40.81)	96	15		65	46		62	49	
Illness or injury in the past 2 weeks				0.542			1.796			0.397
1. Yes	67(24.63)	59	8		39	28		35	32	
2. No	205(75.37)	173	32		100	105		98	107	
Physical health over the past year				6.155**			11.094**			5.213
1. Bad	23(8.46)	17	6		8	15		10	13	
2. Average	145(53.31)	120	25		65	80		63	82	
3. Good	104(38.24)	95	9		66	38		60	44	
Body mass index (BMI)				0.287			1.625			2.847
1. Obese	12(4.41)	11	1		6	6		4	8	
2. Overweight	38(13.97)	36	2		23	15		21	17	
3. Normal weight	175(64.34)	146	29		86	89		82	93	
4. Underweight	47(17.28)	39	8		24	23		26	21	
Physical activity level				2.932			20.573**			12.594**
1. Insufficient	124(45.59)	101	23		49	75		48	76	
2. Adequate	96(35.29)	86	10		50	46		50	46	
3. Active	52(19.12)	45	7		40	12		35	17	
Frequency of daily exercise				5.966			3.698			5.399
1. Less than 1 time per week	41(15.07)	30	11		18	23		16	25	
2. 1–2 times per week	132(48.53)	114	18		63	69		62	70	
3. 3–5 times per week	74(27.21)	66	8		43	31		44	30	
4. 1 time per day	25(9.19)	22	3		15	10		11	14	
Daily eating habits				1.768			4.862			14.378**
1. Not deliberate, spontaneous eating	132(48.53)	111	21		57	75		53	79	
2. Eating out mainly	33(12.13)	28	5		16	17		15	18	
3. Vegetarian dishes and vegetables	26(9.56)	24	2		17	9		16	10	
4. More meat and fewer vegetables	14(5.15)	11	3		8	6		5	9	
5. Meat and vegetables, with a balance of coarse and fine grains	67(24.63)	58	9		41	26		44	23	
Reading type of health education articles				19.217**			28.537**			37.558**
1. Prevention and treatment of infectious diseases	23(8.46)	20	3		12	11		13	10	
2. Prevention and treatment of chronic diseases	5(1.84)	3	2		3	2		2	3	
3. Scientific and health concepts	32(11.76)	28	4		21	11		15	17	
4. Healthy lifestyle and behavior	86(31.62)	76	10		57	29		56	30	
5. Basic health care	12(4.41)	12	0		7	5		8	4	
6. Health knowledge and information	10(3.68)	10	0		6	4		6	4	
7. Safety and first aid	30(11.03)	29	1		12	18		18	12	
8. None	74(27.21)	54	20		21	53		15	59	
Satisfaction with health information				0.459			42.534**			27.248**
1. Not satisfied	34(12.50)	30	4		10	24		10	24	
2. Satisfied	153(56.25)	131	22		61	92		62	91	
3. Very satisfied	85(31.25)	71	14		68	17		61	24	
Academic status				2.767			27.436**			25.05**
1. Not satisfied	67(24.63)	54	13		24	43		23	44	
2. Satisfied	148(54.41)	131	17		69	79		66	82	
3. Very satisfied	57(20.96)	47	10		46	11		44	13	
Interpersonal relationship status				3.013			23.274**			13.434**
1. Not satisfied	51(18.75)	40	11		18	33		18	33	
2. Satisfied	121(44.49)	103	18		51	70		52	69	
3. Very satisfied	100(36.76)	89	11		70	30		63	37	
Self-efficacy level				1.247			19.642**			24.422**
1. Low	60(22.06)	49	11		23	37		18	42	
2. Moderate	122(44.85)	107	15		53	69		53	69	
3. High	90(33.09)	76	14		63	27		62	28	
IDSHLS							12.788**			15.672**
1. High	232(85.29)				129	103		125	107	
2. Low	40(14.71)				10	30		8	32	
eHEALS				12.788**						104.03**
1. High	139(51.10)	129	10					110	29	
2. Low	133(48.90)	103	30					23	110	
HLS-SF12				15.672**			104.03**			
1. High	133(48.90)	125	8		110	23				
2. Low	139(51.10)	107	32		29	110				

*p < 0.05, **p < 0.01.

Unhealthy eating behaviors emerged as the second most prevalent health-risk factor (Figure 2). Furthermore, only 24.63% maintained a balanced diet incorporating coarse and fine grains, meat, and vegetables (Table 2), highlighting that the majority of students exhibit suboptimal dietary habits.

### 3.4 Electronic new media emerging as primary channels for health information acquisition

**Figure 3:**
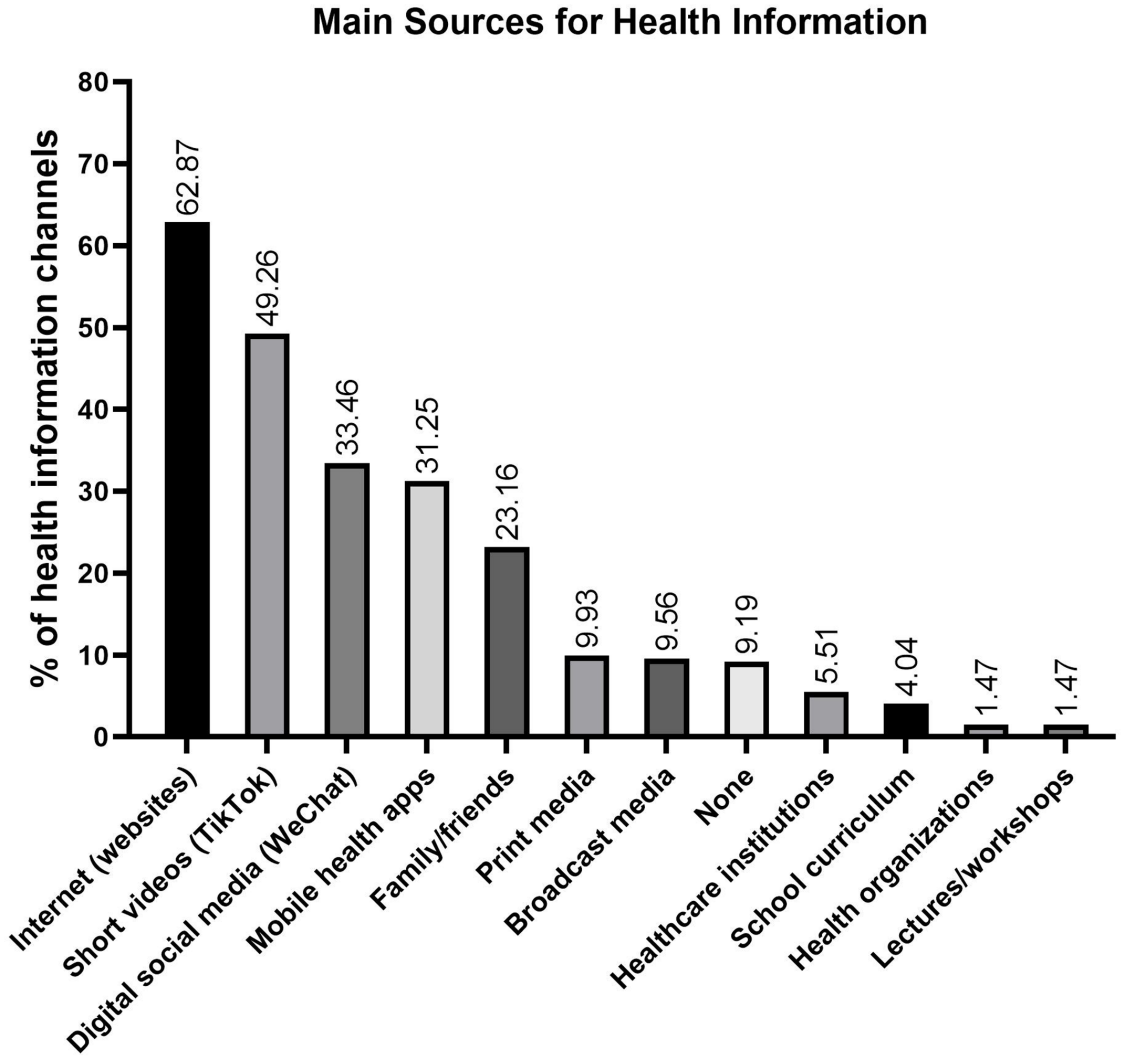
Health information channels among senior high school students. Digital media (internet, short videos, social platforms, mobile health apps) dominate traditional and institutional sources (176.84% vs.11–19.5%).

The quantitative hierarchy of health information sources among senior high school students (see Figure 3) manifests as: internet (62.87%), short video platforms (49.26%), digital social media (33.46%), mobile health apps (31.25%), interpersonal networks (23.16%), print media (9.93%), broadcast media (9.56%), non-engagement (9.19%), healthcare institutions (5.51%), school curricula (4.04%), health organizations (1.47%), and lectures/workshops (1.47%). This transition underscores the displacement of institution-mediated channels (cumulative 11.02%) and traditional media (19.49%) by digital new media (176.84% cumulative frequency).

### 3.5 Variations in levels of IDSHLS, eHEALS, and HLS

The study revealed significant variability in HL levels among senior high school students, with mean scores of 23.23 ± 7.69 for IDSHLS, 28.22 ± 8.10 for eHEALS, and 34.81 ± 10.29 for HLS-SF12 (Table 3). Specifically, 85.29% of students demonstrated high IDSHLS, while 14.71% scored low. For eHEALS, 51.1% were classified as having high literacy, compared to 48.9% with low literacy. Similarly, 48.9% exhibited high HLS-SF12, whereas 51.10% fell into the low category (Table 2). These findings demonstrate the diverse HL levels among students, which may significantly impact their capacity to navigate health-related challenges effectively.

### 3.6 Influencing factors of IDSHLS, eHEALS, and HLS-SF12 in senior high school students

Our study aimed to improve high school students’ health outcomes by elucidating the demographic and behavioral determinants of HL. Initially, we employed Pearson’s chi-square tests to compare the distributions of the IDSHLS, eHEALS, and HLS-SF12 categories across demographic (Table 1) and behavioral (Table 2) characteristics. With the sole exception of home location—which was significantly associated with eHEALS (*χ*^2^ = 11.445, *p* < 0.01) and HLS-SF12 (*χ*^2^ = 5.886, *p* < 0.05)—demographic variables did not yield significant differences (Table 2).

**Table 1:** Demographic characteristics of respondents and the association to IDSHL, eHL, HL (*N* = 272).

Characteristics	*N*(%)	IDSHLS			eHEALS			HLS-SF12		
		≥16.74	<16.74	*X* ^2^	High	Low	*X* ^2^	High	Low	*X* ^2^
Gender				0.105			2.395			0.794
1. Male	150(55.15)	127	23		83	67		77	73	
2. Female	122(44.85)	105	17		56	66		56	66	
Home location				0.084			11.445**			5.886*
1. urban	252(92.65)	214	38		121	131		118	134	
2. rural	20(7.35)	18	2		18	2		15	5	
Stage of study				1.459			0.039			0.473
1. Grade 10	153(56.25)	134	19		79	74		72	81	
2. Grade 11	119(43.75)	98	21		60	59		61	58	
Parental education level				1.549			3.835			0.388
1. Primary school or below	3(1.10)	2	1		3	0		2	1	
2. Middle school	55(20.22)	49	6		31	24		27	28	
3. University degree or above	214(78.68)	181	33		105	109		104	110	
Households disposable annual income				1.261			0.84			0.905
1. Low (30,000 RMB below)	19(6.99)	15	4		9	10		8	11	
2. Medium (30,000–100,000 RMB)	70(25.74)	62	8		39	31		32	38	
3. High (100,000 RMB above)	183(67.28)	155	28		91	92		93	90	

*p < 0.05, **p < 0.01.

Key behavioral factors showed significant associations with HL measures. IDSHLS varied with physical health status (*χ*^2^ = 6.155), health article preferences (*χ*^2^ = 19.217), eHEALS (*χ*^2^ = 12.788), and HLS-SF12 (*χ*^2^ = 15.672; all *p* < 0.01). eHEALS differed by prior infections (*χ*^2^ = 4.172, *p* < 0.05), physical activity (*χ*^2^ = 20.573), health information engagement (reading: *χ*^2^ = 28.537; satisfaction: *χ*^2^ = 42.534), academic/relational status (*χ*^2^ = 27.436/23.274), self-efficacy (*χ*^2^ = 19.642), and both IDSHLS (*χ*^2^ = 12.788) and HLS-SF12 (*χ*^2^ = 104.03; all *p* < 0.01). HLS-SF12 correlated with health behaviors (activity: *χ*^2^ = 12.594; diet: *χ*^2^ = 14.378), information interaction (reading: *χ*^2^ = 37.558; satisfaction: *χ*^2^ = 27.248), psychosocial factors (academic: *χ*^2^ = 25.05; relational: *χ*^2^ = 13.434; self-efficacy: *χ*^2^ = 13.434), and literacy measures (IDSHLS: *χ*^2^ = 15.672; eHEALS: *χ*^2^ = 104.03; all *p* < 0.01) (Table 2).

Subsequent binary logistic regression analysis assessed the impact of these significant factors on high versus low outcomes. Although inter-variable correlations were not significant (data not shown), high IDSHLS was an independent predictor of high HLS-SF12 (OR = 10.281, 95% CI: 1.787–59.139, *p* < 0.01) (Table 4). Moreover, high eHEALS exhibited a strong bidirectional association with high HLS-SF12 (ORs of 23.313 [95% CI: 8.957–60.680] and 23.145 [95% CI: 8.959–59.792], both *p* < 0.01) (Table 4). These results preliminarily demonstrate that, beyond the reciprocal relationship between eHEALS and HLS-SF12, IDSHL independently forecasts enhanced HL.

**Table 4:** Regression analysis of factors influencing IDSHLS, eHEALS, and HLS among senior high school students in Zhengzhou City, 2024.

Dependent variable (experimental group = high)	Independent variable	Regression coefficient	Standard errors	Wald	*P*	Odds ratios (OR)	95% Confidence Interval for OR	
							Upper limit	Lower limit
IDSHL level	eHEALS Level	0.850	0.757	1.260	0.262	2.339	0.530	10.320
	SC-HLS-SF12 Level	2.330	0.893	6.815	0.009**	10.281	1.787	59.139
eHEALS level	IDSHL Level	1.143	0.763	2.242	0.134	3.136	0.703	13.996
	SC-HLS-SF12 Level	3.149	0.488	41.629	0.000**	23.313	8.957	60.680
SC-HLS-SF12 level	IDSHL level	1.440	0.776	3.439	0.064	4.219	0.921	19.320
	eHEALS level	3.142	0.484	42.095	0.000**	23.145	8.959	59.792

**p < 0.01.

## 4 Discussion

This study systematically examined sociodemographic, behavioral, and HL data among high school students, revealing that challenges in emotional, sleep, vision, weight and GI issues as the most pressing health concerns. Notable behavioral risk factors, such as insufficient physical activity and suboptimal dietary habits, further compound these issues, while electronic new media now dominate health information acquisition over traditional media. Moreover, evaluations of IDSHL, eHL, and general HL revealed significant disparities and interrelationships across these domains. These findings necessitate multi-tiered interventions addressing both immediate health risks and foundational literacy gaps. In particular, the observed preliminary synergy between literacy domains and digital-channel information acquisition suggests integrated school programs combining IDSHL pandemic preparedness with eHL digital skill development could enhance health resilience while mitigating health burdens and recurrent infectious disease threats in high-risk subgroups.

Mental health literacy (MHL) is now recognized as a critical determinant within comprehensive health frameworks, particularly for adolescent health outcomes (4). Our data identify emotional distress as the predominant adolescent health burden (prevalence = 52.2%), surpassing conventional morbidity indicators through multisystemic pathophysiological effects (4, 7). Chronic affective dysregulation induces hypothalamic–pituitary–adrenal (HPA) axis hyperactivity, driving glucocorticoid-mediated mitochondrial dysfunction and neuroimmune activation, correlate with cardiovascular risks and gut-brain axis dysregulation (19). School-based interventions combining curricular innovations (e.g., outdoor experiential learning) with circadian-aligned light exposure and microbiota modulation (20), demonstrate co-mitigation of psychological-somatic symptoms, aligning with WHO’s emphasis on structural solutions. Future implementation research must prioritize scalable models that reconcile academic demands with MHL enhancement during adolescence’s critical neuroplastic periods.

Physical inactivity, observed in over 50% of adolescents, remains the most modifiable risk factor in this population. Urbanization and academic pressures contribute to pervasive sedentary behavior, which is a known driver of global all-cause mortality and chronic conditions (21), including cardiovascular, metabolic, vision, sleep, and mental health disorders. Although mechanistic links via the HPA and gut–brain axes have been established (19, 22), meta-analytical evidence suggests that current school-based interventions have achieved limited success in increasing physical activity or reducing sedentary time (23). These insights support the implementation of whole-school strategies, such as structured exercises, to foster sustainable behavioral change and mitigate long-term health burdens associated with inactivity.

Unbalanced dietary patterns ranked second in terms of health-risk behaviors, with approximately 40% of students displaying suboptimal dietary patterns and only one-quarter meeting adequate nutritional thresholds. Diet and nutrition impacting on the microbiome via gut–brain crosstalk and metabolic–endocrine interactions modulate health status and various diseases, including cancer, diabetes, obesity, chronic inflammatory diseases, mood disorders (e.g., anxiety and depression), neuropsychiatric disorders, GI disorders and cardiopneumatic disorders (24). Recent evidence suggests that adverse dietary habits impair neurodevelopment by altering gut microbiota, which influences neurotransmitter activity, neuroinflammation, and blood–brain barrier integrity (25). These observations underscore the importance of childhood nutrition in shaping brain morphology and long-term health trajectories. Consequently, comprehensive, school-based dietary and physical activity programs that integrate balanced nutrition with structured health curricula are warranted.

Our study identifies critical behavioral gaps among adolescents, with only 34% demonstrating comprehensive risk avoidance and 2.57% engaging in unsafe sexual practices—findings aligned with China CDC epidemiological trends showing HIV cases in 15–19-year-olds rising from 15.7% (2010) to 24% (2019), predominantly via unprotected intercourse (26). While WHO-endorsed comprehensive sexuality education (CSE) programs effectively enhance the sexual health of young people (<25 years) in high-risk populations (27), systematic reviews reveal improvements in adolescents’ knowledge and attitudes do not consistently translate into long-term behavioral change (28). Thus, it is urgent that integrating targeted digital interventions with school-based CSE frameworks to bridge the knowledge–behavior gap in sexual health prevention strategies.

Survey data indicate a significant shift in health information acquisition among senior high school students, with electronic new media displacing traditional channels. Figure 3 shows that the internet (62.87%), short video platforms (49.26%), digital social media (33.46%), and mobile health apps (31.25%) account for a cumulative utilization frequency of 176.84%, vastly exceeding that of traditional media (19.49%) and institutional sources (11.02%). This trend highlights three points: (i) digital platforms are supplanting institution-mediated channels; (ii) peer-generated content dominates expert-curated material, necessitating the integration of evidence-based information; and (iii) the 6.8:1 utilization ratio emphasizes the need for robust eHL. Thus, paradigm-shifting interventions that enhance eHL via electronic media are essential for a contemporary health education framework (2, 16).

Our findings demonstrate notable methodological consistency through cross-study validation, revealing persistent HL challenges. The mean eHEALS score (28.22 ± 8.10) surpasses Japanese non-medical students (23.1 ± 6.7) (14) yet trails undergraduate populations (30.16 ± 6.31) (17), while aligning closely with Japanese medical trainees (27.0 ± 6.6) (14) and Guo et al.’s (18) cohort (28.58 ± 7.00) – collectively suggesting stagnant digital health competency despite exponential technological growth. This pattern persists in IDSHL metrics: our average (23.23 ± 7.69) mirrors Tian et al.’s (15) national 15–69-year-old population (22.07 ± 7.91), including senior high schoolers (23.85 ± 6.76), confirming minimal pandemic-era improvements over the past decade. Notably, our HLS-SF12 score (34.81 ± 10.29) marginally exceeds Sun et al.’s (3) general population benchmark (34.31 ± 8.40), validating both population reliability and the 42-item multidimensional scale’s utility in adolescent HL assessment. These cross-temporal and cross-population comparisons expose systemic health education failures, particularly in digital HL and infectious disease preparedness, necessitating urgent curriculum reforms integrating evidence-based pedagogical innovations.

Longitudinal national surveillance reveals persistent IDSHL deficits in China, with only 29.3% demonstrating adequate knowledge versus 61.3% safety/first aid competency, 56.3% scientific health understanding, and 44.0% general HL (29). While high school students show reduced low-IDSHL prevalence (14.7%) compared to Tian’s benchmark (23.8%) (15), stagnant mean scores and bimodal distribution – epidemic cycles may temporarily boost literacy in some adolescents, rapid test completion and non-response in disengaged subgroups depress overall performance. Only 8.5% express interest in epidemiological topics, reflecting cognitive prioritization of immediate personal risks over abstract public health threats – a bias amplified by chronic disease-focused infodemic narratives (30). Curricular deficiencies in zoonotic transmission mechanisms compound these disparities, necessitating AI-enhanced, digital curricular of pathogen literacy and rigorous, longitudinal evaluation of outbreak-responsive behaviors (31).

Although sociodemographic variations were minimal, significant behavioral differences underscore the necessity of incorporating these factors into precision intervention frameworks. While regression analyses in this study did not consistently achieve statistical significance—likely due to sample size constraints—larger studies have confirmed that sociodemographic and behavioral factors exert measurable influences on HL outcomes (15, 17), necessitating their consideration in intervention design. Notably, variables showing *χ*^2^ significance failed to persist in regression analyses, reflecting methodological limitations rather than null relationships. These patterns underscore the heightened sample size requirements and multiple comparison adjustments inherent to multivariate regression frameworks.

A notable finding is the interdependent reinforcement among eHL, general HL, and IDSHL, wherein technology-mediated competencies enhance general HL efficacy during outbreaks (14, 17). This speculative interaction aligns with emerging evidence showing integrated literacies optimize context-specific health behaviors in digital epidemiological contexts. As demonstrated by Li et al. (17), university students with elevated eHLS and HLS exhibit heightened adherence to COVID-19 protocols through improved navigation of digital epidemiological data. Crucially, eHLS’s stronger behavioral association versus HLS reflects how digital platforms structurally contextualize outbreak information, refining risk perception to mitigate transmission in technology-dependent populations. These insights mandate equity-focused strategies addressing technological access disparities and cognitive preparedness gaps, particularly in low-HLS demographics. This transition from isolated competencies to integrated literacy ecosystems could redefine preparedness frameworks for hyperconnected societies.

While this study providing foundational insights, limitations include self-report biases, measurement heterogeneity and a single-site cross-sectional design constraining causal inference and generalizability, particularly given adolescents’ evolving lifestyles and social desirability. Homogeneous single-school sampling limits socioeconomic diversity, exacerbated by underrepresentation of international students due to institutional constraints. The study’s generalizability and causal interpretation of findings remain constrained by cross-sectional design, sample size and regional sampling characteristics, with findings constituting preliminary evidence requiring validation through large-scale longitudinal studies prior to broader application. Reliance on three distinct 42-item scales rather than a unified instrument may fragment HL assessment. Future investigations should employ multisite longitudinal designs integrating objective biomarkers and socioeconomic diversity, develop validated Digital Health Literacy Instruments (DHLI) with infectious disease modules, and implement blended interventions combining digital platforms (such as AI and various social medias) with institutional surveillance systems for personalized eHealth education.

In summary, this study reveals significant disparities in health concerns, risk behaviors, health information acquisition, and literacy levels of IDSHL, eHL, and general HL among high school students, suggesting their interconnected and mutually reinforcing nature. The preliminary findings stress the urgency of integrated interventions targeting behavioral risks and literacy deficits, particularly under persistent infectious disease threats. Crucially, holistic strategies combining emotional well-being support, physical activity promotion, and nutritional guidance must align with targeted digital and pathogen literacy enhancement initiatives to mitigate multidimensional health risks. Such coordinated strategies hold promises for empowering adolescents to adopt sustainable health behaviors and fostering health-literate communities amid advancing digital health innovations and persistent health challenges.

## Data Availability

The original contributions presented in the study are included in the article/Supplementary material, further inquiries can be directed to the corresponding author.
