# Supplementary material for: Associations of infectious disease-specific, electronic, and general health literacy among high school students with prevalent health challenges: a cross-sectional study
Source: Front Public Health. 2025 May 13;13:1613375. doi: 10.3389/fpubh.2025.1613375 (PMC12117663; doi:10.3389/fpubh.2025.1613375)
Supplement: Supplementary file 1 [file Data_Sheet_1.PDF]

Health Literacy Questionnaire for High School Students

Dear High School Students,

We invite you to participate in an anonymous survey designed to assess your health literacy. All responses will be kept strictly confidential, and the data collected will contribute to improving future health outcomes across broader populations. Please answer each question honestly based on your personal experiences and perspectives. We appreciate your participation.

Background and Objectives

Health literacy is defined as an individual's ability to access, understand, process, and use essential health information and services to make informed decisions about personal well-being. Research has demonstrated that health literacy is a critical determinant of health outcomes. This survey aims to evaluate current levels of health literacy and to inform the development of educational interventions that promote disease prevention and overall health.

Procedure

The survey is conducted anonymously. You will be asked to provide basic demographic information (without including personally identifiable data) and to respond to questions regarding your health attitudes and behaviors. The estimated time for completion is 6 to 10 minutes.

Risks

Although some questions related to health habits, attitudes, or behaviors may cause mild discomfort, there are no significant risks associated with participation. You are free to withdraw from the survey at any time without penalty.

Benefits

Participation may help you gain insights into common health behaviors and contribute to a preliminary self-assessment of your health literacy. Moreover, your responses will assist in shaping strategies for enhancing public health education and interventions.

Confidentiality

All responses will be anonymized and securely stored. No personally identifiable information will be collected.

Voluntary Participation

Your participation in this survey is entirely voluntary, and you may withdraw at any stage without the need to provide a reason.

Informed Consent

By submitting this questionnaire, you confirm that you have read and understood the information provided, consent to participate voluntarily, and acknowledge your right to withdraw at any time.

For further inquiries, please contact the **Boya Health Voluntary Club** via WeChat at GY15838332542.

Section 1: Demographic Information

1. **Date: ____________________________**
2. **Gender:**
   A. Male
   B. Female
3. **Ethnicity:**
   A. Han Chinese
   B. Other
4. **Residential Area:**
   A. Urban
   B. Rural
5. **Grade Level:**
   A. Grade 10
   B. Grade 11
6. **Parental Education Level:**
   A. Primary School or Below
   B. Secondary School (including vocational training)
   C. College/University or Higher
7. **Annual Disposable Household Income (RMB):**
   A. Low (<30,000)
   B. Medium (30,000–100,000)
   C. High (>100,000)
8. **Daily Internet Usage:**
   A. Less than 1 hour
   B. 1–2 hours
   C. More than 2 hours

Section 2: Health Status

9. **In the past year, have you contracted a communicable disease?**
   A. Yes
   B. No
10. **During the past two weeks, have you sought medical care, taken medication, or missed school due to illness or injury?**
   A. Yes
   B. No

11. **How would you rate your health status over the past year?**
    A. Poor (1 point)
    B. Average (2 points)
    C. Good (3 points)
12. **In the past year, what health concerns have you experienced? (Select between 1 and 5)**
    A. Emotional issues (e.g., anxiety, depression)
    B. Weight-related concerns (e.g., obesity, underweight)
    C. Sleep disturbances (e.g., insomnia, inadequate sleep)
    D. Vision problems (e.g., decline in visual acuity)
    E. Oral health issues (e.g., toothache, bleeding gums)
    F. Gastrointestinal problems (e.g., diarrhea, abdominal pain)
    G. Respiratory issues (e.g., frequent colds, cough)
    H. Skin
    I. Chronic diseases (e.g., hypertension, diabetes)
    J. Gynecological issues (e.g., menstrual pain, infections)
    K. Dizziness, Headache
    L. Muscle soreness, Fatigue
    M. Worried about possibly having a mental or psychological disorder
    N. Concerned about potentially suffering from organic diseases or sudden death
    O. None

Section 3: Health Behaviors

13. **Which health risk behaviors have you engaged in? (Select between 1 and 5)**
    A. Intentional injury (e.g., fighting, self-harm)
    B. Unintentional injury (e.g., accidents, poisoning)
    C. Substance abuse (e.g., tobacco, alcohol, drugs)
    D. Behavioral addictions (e.g., gaming, gambling)
    E. Unsafe sexual practices
    F. Unhealthy diet (e.g., excessive fat/sugar intake)
    G. Physical inactivity
    H. None
14. **What is your Body Mass Index (BMI** calculated by dividing weight (in kilograms) by the square of height (in meters).) **classification?**
    A. Obese (BMI ≥ 28.0)
    B. Overweight (BMI 24.0–27.9)
    C. Normal (BMI 18.5–23.9)
    D. Underweight (BMI < 18.5)

15. **According to the Chinese Guidelines (2021)** (≥60 minutes of daily moderate-to-vigorous physical activity. Incorporate muscle- and bone-strengthening exercises ≥3 days weekly. Minimize sedentary periods (≤1 hour per session) and restrict total daily screen time to <2 hours), **what is your physical activity level?**
    1. Insufficient
    2. Adequate
    3. Active
16. **Which exercise modalities do you participate in? (Select between 1 and 3)**
    A. Walking
    B. Jogging
    C. Cycling
    D. Racquet sports
    E. Aerobics
    F. Soccer
    G. Basketball
    H. Weight training
    I. Dancing
    J. Swimming
    K. Other
    L. Rarely exercise
17. **How frequently do you engage in moderate-to-vigorous physical activity (≥60 minutes per session)?**
    A. Less than 1 session per week
    B. 1–2 sessions per week
    C. 3–5 sessions per week
    D. Daily
18. **Which best describes your dietary habits, based on the Chinese Dietary Guidelines (2022)?**
    A. Unstructured eating
    B. Reliance on snacks/takeout (high in salt/oil/sugar)
    C. Plant-based, low in salt/oil/sugar
    D. Meat-heavy, low in fiber
    E. Balanced diet with moderate nutrient intake

Section 4: Health Information

19. **What are your primary interests regarding health information?**
    A. Nutrition
    B. Exercise

C. Mental health
D. Skincare
E. Infectious diseases
F. Chronic diseases
G. Health policies
H. Other
I. No interest

20. **What are your main sources for obtaining health information? (Select between 1 and 3)**
A. Broadcast media
B. Print media
C. Internet (websites, search engines)
D. Mobile health apps
E. Digital social media (e.g., WeChat)
F. Short videos (e.g., TikTok)
G. Family/friends
H. Healthcare institutions
I. Health organizations
J. Lectures/workshops
K. School curriculum
L. Other
M. None

21. **Which topics do you prefer for health education?**
A. Infectious disease prevention
B. Chronic disease management
C. Scientific health concepts
D. Healthy lifestyles (e.g., exercise, nutrition)
E. Medical care
F. General health knowledge
G. Safety/first aid
H. Other
I. None

22. **How satisfied are you with your access to health information?**
1. Dissatisfied
2. Satisfied
3. Very Satisfied

Section 5: Self-Assessment

23. **How satisfied are you with your academic performance?**
    1. Dissatisfied
    2. Satisfied
    3. Very Satisfied
24. **How satisfied are you with your interpersonal relationships?**
    1. Dissatisfied
    2. Satisfied
    3. Very Satisfied
25. **How would you rate your self-efficacy (i.e., your confidence in achieving your goals)?**
    1. Low
    2. Moderate
    3. High

Section 6: Infectious Disease Prevention Literacy

Please select one response for each statement.

26. **Antibiotics are effective in treating hepatitis B.**
    A. Agree
    B. Disagree
    C. Unsure
27. **Boiled lamb is safe to consume when its color changes from dark to pale.**
    A. Agree
    B. Disagree
    C. Unsure
28. **Hepatitis B can be transmitted via sexual contact.**
    A. Agree
    B. Disagree
    C. Unsure
29. **Tuberculosis is considered cured if symptoms resolve after two weeks of treatment.**
    A. Agree
    B. Disagree
    C. Unsure
30. **Consuming cooked poultry that died from disease is safe.**
    A. Agree
    B. Disagree
    C. Unsure
31. **HIV-infected individuals exhibit visible physical symptoms.**

A. Agree

B. Disagree

C. Unsure

32. **Young adults are immune to tuberculosis due to robust physical health.**

A. Agree

B. Disagree

C. Unsure

33. **Vaccination is unnecessary for indoor pets that rarely go outside.**

A. Agree

B. Disagree

C. Unsure

34. **Restricting the mobility of individuals with infectious diseases violates human rights.**

A. Agree

B. Disagree

C. Unsure

35. **Antibiotics should be administered immediately for the treatment of influenza.**

A. Agree

B. Disagree

C. Unsure

Multiple Choice Questions (Select one answer for each):

36. **The term "H1N1" refers to:**

A. Hepatitis A

B. A therapeutic drug

C. Influenza A

D. Unsure

37. **Food service workers must obtain:**

A. An employment permit

B. A health certificate

C. A temporary residency permit

D. Unsure

38. **"Contaminated water" refers to:**

A. Domestic wastewater

B. Water polluted by pathogens

C. Water from epidemic zones

D. Unsure

39. **The "sugar pill" vaccine is used to prevent:**

A. Poliomyelitis
B. Tuberculosis
C. Measles
D. Unsure

40. **HIV is classified as:**
A. A bacterium
B. A virus
C. A parasite
D. Unsure

41. **Primary symptoms suggestive of hand-foot-mouth disease include:**
A. Cough and sputum production
B. Rashes on the hands, feet, or oral mucosa
C. Excessive thirst and urination
D. Unsure

42. **The high-risk population for hand-foot-mouth disease is:**
A. Preschool children
B. Adolescents
C. Adults
D. Unsure

43. **Vaccines are defined as:**
A. Therapeutic biologics
B. Antibiotics
C. Preventive biologics
D. Unsure

44. **Hepatitis B transmission routes include:**
A. Sharing toothbrushes
B. Sharing utensils
C. Handshaking
D. Unsure

45. **The recommended immediate action after a minor animal bite is:**
A. Rinsing with water only
B. Washing with soap and obtaining rabies vaccination
C. Using folk remedies
D. Unsure

46. **For a nail puncture injury, the recommended vaccination is:**
A. DPT vaccine
B. Tetanus vaccine
C. Rabies vaccine

D. Unsure

47. **Common outdoor mosquito breeding sites include:**
    A. Stagnant water containers
    B. Grasslands or forests
    C. Soil in wild areas
    D. Unsure

Section 7: eHEALS Health Literacy Scale

Please indicate your level of agreement with each statement using a 5-point Likert scale (1 = Strongly Disagree, 5 = Strongly Agree):

48. I can locate credible health resources online. **Score: _____**
49. I use online information to address health concerns. **Score: _____**
50. I understand the various types of health information available online. **Score: _____**
51. I am aware of reliable online platforms for obtaining health information. **Score: _____**
52. I apply online health resources to enhance my well-being. **Score: _____**
53. I can evaluate the quality of online health information. **Score: _____**
54. I can distinguish between high- and low-quality online health content. **Score: _____**
55. I am confident in using online information to make health-related decisions. **Score: _____**

Section 8: Simplified Chinese Health Literacy Scale (SC-HLS-SF12)

Please rate the difficulty of each task on a 4-point scale (1 = Very Difficult, 4 = Very Easy):

56. Accessing treatment information for a diagnosed condition. **Score: _____**
57. Understanding medication instructions. **Score: _____**
58. Comparing the pros and cons of treatment options. **Score: _____**
59. Calling an ambulance in emergencies. **Score: _____**
60. Finding strategies for managing mental health. **Score: _____**
61. Understanding the purpose of health screenings. **Score: _____**
62. Determining the required vaccinations. **Score: _____**
63. Implementing disease prevention advice from peers. **Score: _____**
64. Identifying activities that benefit mental health. **Score: _____**
65. Interpreting health-related media content. **Score: _____**
66. Assessing the impact of daily habits on health. **Score: _____**
67. Enrolling in fitness programs when motivated. **Score: _____**
